# Supplementation with Red Palm Oil Increases *β*-Carotene and Vitamin A Blood Levels in Patients with Cystic Fibrosis

**DOI:** 10.1155/2015/817127

**Published:** 2015-01-26

**Authors:** Olaf Sommerburg, Silke De Spirt, Annett Mattern, Cornelia Joachim, Claus-Dieter Langhans, Kalanithi Nesaretnam, Werner Siems, Wilhelm Stahl, Marcus A. Mall

**Affiliations:** ^1^Division of Pediatric Pulmonology & Allergy and Cystic Fibrosis Centre, Department of Pediatrics III, Children's Hospital, University of Heidelberg, Im Neuenheimer Feld 430, 69120 Heidelberg, Germany; ^2^Translational Lung Research Centre Heidelberg (TLRC), Member of the German Centre for Lung Research (DZL), Im Neuenheimer Feld 350, 69120 Heidelberg, Germany; ^3^Institute of Biochemistry and Molecular Biology I, Faculty of Medicine, Heinrich-Heine-University Dusseldorf, 40001 Dusseldorf, Germany; ^4^Division of Metabolic Diseases and Newborn Screening Centre, Department of Paediatrics I, Children's Hospital, University of Heidelberg, Im Neuenheimer Feld 430, 69120 Heidelberg, Germany; ^5^Product Development and Advisory Services, Malaysian Palm Oil Board (MPOB), 6 Persiaran Institusi, Bandar Baru Bangi, 43000 Kajang, Selangor, Malaysia; ^6^Research Institute of Physiotherapy and Gerontology, KortexMed Institute of Medical Education, Hindenburgring 12a, 38667 Bad Harzburg, Germany; ^7^Department of Translational Pulmonology, University of Heidelberg, Im Neuenheimer Feld 350, 69120 Heidelberg, Germany

## Abstract

Patients with cystic fibrosis (CF) show decreased plasma concentrations of antioxidants due to malabsorption of lipid soluble vitamins and consumption by chronic pulmonary inflammation. *β*-Carotene is a major source of retinol and therefore is of particular significance in CF. The aim of this study was to investigate the effect of daily intake of red palm oil (RPO) containing high amounts of *β*-carotene on the antioxidant levels in CF patients. Sixteen subjects were recruited and instructed to enrich their food with 2 to 3 tablespoons of RPO (~1.5 mg of *β*-carotene) daily over 8 weeks. Carotenoids, retinol, and *α*-tocopherol were measured in plasma at baseline and after intervention. In addition *β*-carotene, lycopene, *α*-tocopherol, and vitamin C were measured in buccal mucosa cells (BMC) to determine the influence of RPO on antioxidant tissue levels. Eleven subjects completed the study properly. Plasma *β*-carotene, retinol, and *α*-carotene of these patients increased, but plasma concentrations of other carotenoids and *α*-tocopherol as well as concentrations of *β*-carotene, lycopene, *α*-tocopherol, and vitamin C in BMC remained unchanged. Since RPO on a daily basis did not show negative side effects the data suggest that RPO may be used to elevate plasma *β*-carotene in CF.

## 1. Introduction

In healthy people reactive oxidant species are controlled by a number of enzymatic and nonenzymatic antioxidants [1]. Approximately 85–90% of patients with cystic fibrosis (CF) suffer from pancreatic insufficiency (PI), which predisposes them to malabsorption of lipids and lipid soluble vitamins leading to a deficiency of nonenzymatic antioxidants [2]. Chronic pulmonary infection of CF patients results in a chronic activation of immune cells, especially neutrophilic granulocytes, leading to a permanent production of free radicals and lipid peroxidation (LPO) products, thus triggering inflammation [3–6]. As a result, antioxidants are permanently used up in CF patients to respond to increased oxidative stress leading to a depletion of these micronutrients [7–9]. The nutritional status of CF patients is recognized as strong predictor of the severity of CF lung disease. In order to overcome PI, a high energy intake, pancreatic enzyme replacement therapy, and supplementation of the lipid soluble vitamins A, D, E, and K have become standard of care (e.g., [10, 11]).

Carotenoids are lipid soluble micronutrients which were reported to be low in the plasma of CF patients (e.g., [12, 13]). *β*-Carotene is one of more than 50 carotenoids present in the human diet and is known as provitamin A. *β*-Carotene is seen as the major plant source of retinol. Apart from that, *β*-carotene may act as antioxidant and is an important singlet-oxygen-scavenger in the human body [14]. In a number of studies, *β*-carotene supplementation in CF patients led to positive effects such as increase of total antioxidant capacity and decrease of pulmonary exacerbations [15–17]. Although the role of carotenoids in inflammation and in the pathogenesis of CF is not completely understood, it can be assumed that a low status of carotenoids has harmful effects such as those seen also for other antioxidants. The major source of carotenoids is fruits and vegetables. However, an alternative source rich in *β*-carotene, *α*-carotene, and tocotrienols (the latter have antioxidant properties similar to tocopherols) is red palm oil (RPO) that is produced in areas of Africa and Southeast Asia and believed to have beneficial health effects [18, 19]. Since daily food can be easily enriched with RPO as shown in previous studies [20, 21], we speculated that RPO may be a good source of *β*-carotene for CF patients. The aim of this pilot study was to determine if *β*-carotene blood levels in CF can be elevated using RPO in the daily diet. Second, it was investigated if an increase in plasma *β*-carotene concentrations by RPO supplementation modulates blood levels of retinol, *α*-tocopherol, and other carotenoids. Finally, we investigated if an increase of plasma *β*-carotene has effects on tissue concentrations of antioxidants by measuring *β*-carotene, lycopene, *α*-tocopherol, and vitamin C in buccal mucosa cells (BMC) in patients with CF.

## 2. Materials and Methods

### 2.1. CF Patients

Between November 2009 and June 2010 16 CF patients from the CF Centre Heidelberg, Germany, were recruited to take part in the study and 11 of them completed the study successfully. 20 CF patients were primarily proven for eligibility if they fulfilled the following criteria: presence of CF disease with pancreatic insufficiency, age ≥ six years, FEV1 ≥ 30%, no hospitalizations because of pulmonary exacerbations six weeks prior to recruitment, absence of severe liver disease as defined by clinical findings of portal hypertension, liver cirrhosis, or liver enzymes AST or ALT > 2× of the upper limit of normal. Subjects were also excluded if they had diabetes mellitus requiring insulin therapy or significant anaemia (Hb ≤ 9 mg/dL) or in case of pregnancy. Oral supplementation of vitamins A (retinol), D, E, and K prior to and during the study was allowed according to the daily dosages recommended by the European or US American consensus guidelines [10, 11]. CF patients were excluded from the study if they had oral supplementation of *β*-carotene six months prior to the study or had participated in another interventional clinical trial within 90 days prior to the first visit. A summary of patient demographics is provided in Table 1.

The study was approved by the Ethical Committee of the Medical Faculty of the University of Heidelberg (S-175/2007). Informed written consent was obtained from each participating subject older than 18 years. For subjects between six and 18 years of age an age-appropriate consent was obtained in addition to the written consent signed by the subject's parents or legal guardian.

### 2.2. Intervention with Red Palm Oil

The RPO used in that study was kindly provided from the Malaysian Palm Oil Board which is an agency of the Malaysian government entrusted to promote the use of palm oil products. RPO is edible oil rich in *β*-carotene and *α*-carotene. The whole carotenoid composition of RPO was published elsewhere [22] and is given in Table E1 in the Supplementary Material (available online at http://dx.doi.org/10.1155/2015/817127). The daily intake within this pilot study was about ~1.5 mg of *β*-carotene which corresponds to approximately 30 mL of RPO (~2-3 tablespoons). Considering RPO as mixed food this amount of *β*-carotene and also the amount of *α*-carotene would correspond at least to 162 retinol activity equivalents [23]. Participants were advised that at least two-thirds of the oil should be used unheated, for example, in salads. Less than one-third of the oil was allowed to be used for cooking or frying. The study subjects received at least 1.5 Litres of RPO and could request more, if necessary for any reason, to ensure sufficient amount of RPO for the study period. The intake of the oil and possible side effects had to be protocolled daily in a patient diary. The duration of intervention with RPO was eight weeks.

### 2.3. Measurement of Antioxidants

Measurements of vitamins, carotenoids, and clinical parameters were performed at baseline and at the end of the RPO intervention. For analysis of antioxidants, blood samples were collected in heparinized tubes. The samples were centrifuged immediately and stored frozen at −80°C until analysis. Carotenoids (lutein, zeaxanthin, cryptoxanthin, lycopene, *α*-carotene, and *β*-carotene) were analysed by HPLC with UV/Vis detection with a second UV/Vis detector connected in series for quantitation of retinol (vitamin A) and *α*-tocopherol (vitamin E) [24, 25].

BMC of patients were taken at baseline and 8 weeks after start of RPO supplementation at the same time points as blood samples. *β*-Carotene, lycopene, *α*-tocopherol, and vitamin C of the BMC samples were determined by BioTeSys GmbH (Esslingen, Germany) using an accredited method according to DIN EN ISO/IEC 17025 as previously described [26]. In summary, after cleaning the oral cavity, a tooth brush was used to obtain mucosa cells by wiping 25 times over each side of the buccal epithelium. Next, the tooth brush was rinsed in buffer; the proteins of the obtained cells were precipitated and extracted. After vigorous shaking and centrifugation the supernatant was used for analyses. For storage, the samples were frozen at −80°C. After thawing samples were analysed by RP-HPLC using an HPLC system (Waters Corporation, Milford MA, USA). Ascorbic acid was quantified using a coulometric detector (Coulochem II, ESA Inc., Chelmsford MA, USA), *α*-tocopherol was analysed by fluorometric detection (2475 Multi-Wavelength Fluorescence Detector, Waters Corporation, Milford MA, USA), and the carotenoids were detected by UV-Vis absorption (2487 UV/Visible Detector, Waters Corporation, Milford MA, USA). BMC antioxidant content was given per *μ*g DNA, after the DNA content of the cells was determined by a diphenylamine assay [27].

### 2.4. Data Analysis

All data of the study subjects are presented as single values at baseline and after intervention by RPO supplementation. A sample size calculation was done before the pilot study. Based on earlier observations we expected a minimal difference in means of 0.08 *μ*mol/L and a standard deviation of 0.08 before and after intervention with RPO. Considering a power of 80% and an alpha of 0.05 a sample size of 10 would have been necessary to show statistical differences (calculated with SigmaStat 4.0, Systat Software, San Jose, CA, USA). Statistical comparisons were performed with Wilcoxon matched-pairs signed rank test for nonparametric data. Statistical significance was defined as *P* < 0.05 and statistical analysis was performed with the GraphPad Prism 6 software (GraphPad Software, La Jolla, CA, USA).

## 3. Results and Discussion

Twenty CF patients at our CF Centre fulfilled the inclusion criteria of the study. Sixteen of these CF patients agreed to participate in the study and 13 patients completed eight weeks of RPO supplementation. One CF patient stopped RPO intake already after three weeks because of personal reasons. Two other subjects had to be excluded from the study because of pulmonary exacerbations during the intervention time. Eleven of the 13 subjects who completed the study had taken the required daily amount of 2 to 3 tablespoons of RPO and had documented the intake properly. The other two had a poor documentation of their RPO intake and were, therefore, excluded from evaluation. The demographics and clinical characteristics of the 16 CF patients who entered the study and of the 11 CF patients who were eligible for evaluation are given in Table 1.

**Figure 1:**
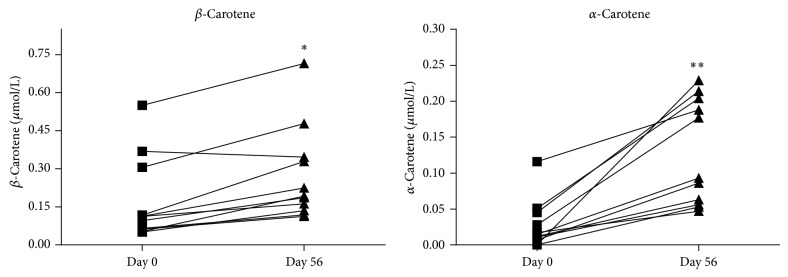
Change of plasma concentration of *β*-carotene and *α*-carotene in the blood of CF patients after eight weeks of supplementation with red palm oil. Values are given in *μ*mol/L. Concentrations of both *β*-carotene and *α*-carotene were significantly increased (day 56) compared with baseline (day 0) (^*^ *P* < 0.002, ^**^ *P* < 0.001).

At baseline, plasma carotenoid concentrations of all 11 CF patients were measured in a low range. The data are in agreement with previous reports that showed decreased carotenoid plasma concentrations in patients with CF when compared to healthy controls [7, 13, 28]. CF patients are at risk of developing a low status of plasma carotenoids due to PI and consecutive malabsorption of lipid soluble micronutrients as well as increased antioxidant need due to chronic inflammation and chronic lung infection. A few previous studies demonstrated beneficial effects of *β*-carotene supplementation in CF patients [15–17]. Patients supplemented with *β*-carotene (1 mg/kg body weight over 24 weeks) showed decreased markers of oxidative stress and less pulmonary exacerbations compared to patients taking placebo [16]. These positive effects may be related to the antioxidant function of *β*-carotene as well as to its function as provitamin A. In third-world countries *β*-carotene is known to be a very important source of retinol. For that reason, a number of *β*-carotene supplementation studies were carried out in developing countries in Africa and Southeast Asia. One approach to supplement *β*-carotene safely in children and pregnant women was done by using RPO. In these studies it was shown that consumption of RPO enriched food led to increased plasma retinol levels [20, 21, 29]. In our pilot study, RPO was used for the first time to supplement *β*-carotene in CF patients. We show that using RPO in food over eight weeks led to increased plasma concentrations of *β*-carotene in all but one of our 11 CF patients (Figure 1). Further, *α*-carotene was increased in all 11 CF patients after RPO supplementation (Figure 1). The changes of *β*-carotene and *α*-carotene from baseline were statistically significant (*P* < 0.002 and *P* < 0.001, resp.). Notably, previous supplementation studies in CF patients used *β*-carotene doses of 1 mg/kg body weight to raise plasma *β*-carotene levels [15–17]. It is therefore noteworthy that we obtained an increase in plasma *β*-carotene and retinol levels with an RPO supplementation regimen corresponding to *β*-carotene doses as little as ~1.5 mg total per day (range of 0.02 to 0.05 mg/kg body weight in our patients). These results suggest that RPO may represent an ideal source for *β*-carotene for CF patients with PI.

**Figure 2:**
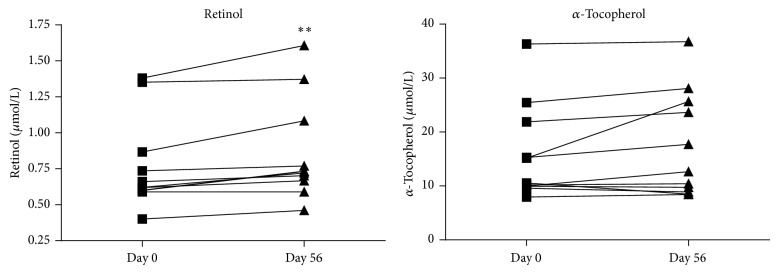
Plasma concentrations of retinol and *α*-tocopherol at baseline and after RPO supplementation. Values are given in *μ*mol/L. Concentration of retinol was significantly increased (day 56) when compared with baseline (day 0) (^**^ *P* < 0.001). Concentration of *α*-tocopherol remained unchained compared with baseline.

**Figure 3:**
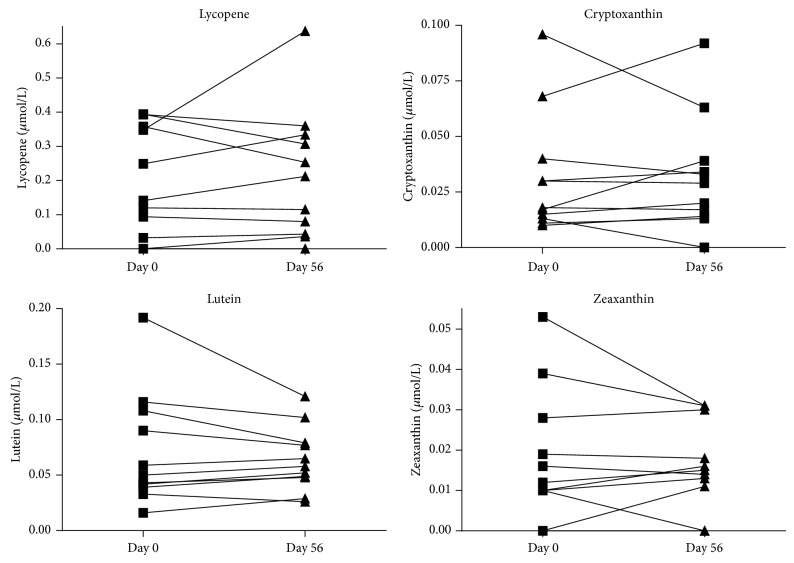
Plasma concentration of lycopene, cryptoxanthin, lutein, and zeaxanthin at baseline and after RPO supplementation. Values are given in *μ*mol/L. Concentrations of lycopene, cryptoxanthin, lutein, and zeaxanthin did not change compared with baseline.

Intervention with RPO also increased plasma retinol in all of the 11 CF patients. The changes were statistically significant (*P* < 0.001, Figure 2). Notably, this result was seen despite the parallel supplementation of vitamin A which was administered continuously to maintain vitamin A plasma values in the normal range. One explanation for that result might be that *β*-carotene from RPO may have a much better bioavailability than vitamin A from supplements. Plasma concentrations of *α*-tocopherol (vitamin E) and the carotenoids lycopene, cryptoxanthin, zeaxanthin, and lutein did not change under RPO supplementation (Figures 2 and 3). This result is important since few studies reported that supplementation of *β*-carotene in high dosages (e.g., 20 mg/day for 6.7 years [30] or 100 mg/day for 6 days [31]) may lead to depletion of other carotenoids in the plasma. However, beside *β*-carotene, these other carotenoids are also of importance because of their particular functions [32]. Two examples are lutein and zeaxanthin which were also shown to be diminished in CF patients [33]. As a consequence, affected CF patients were reported to show a significantly decreased macular pigment density in the retina [33]. This effect is considered to be clinically relevant because lutein, its metabolite mesozeaxanthin, and zeaxanthin are localized in different regions of the human retina serving as optical filter and antioxidant to protect the retinal tissue [34, 35].

To determine the impact of increased *β*-carotene plasma levels on tissue concentrations, we used highly sensitive methods to quantify *β*-carotene, lycopene, *α*-tocopherol, and vitamin C in BMC. In most samples only low concentrations of these antioxidants could be detected in BMC and some samples showed concentrations below the detection limit. A summary of these results is given in Table 2. In a previous study, Back et al. [13] measured antioxidant concentrations in BMC of CF patients. They showed in their population an increase of *β*-carotene measured in BMC after *β*-carotene supplementation that was paralleled by a decrease of oxidative stress markers [13]. In the present study, we did not find an effect on the concentrations of *β*-carotene or the other antioxidants after intervention with *β*-carotene containing RPO enriched food. We speculate that this lack of effect on tissue antioxidant levels in our study may be explained by the limited number of patients, the low dose, and the duration of the *β*-carotene supplementation in our study.

Our pilot study has some limitations. First, we had no placebo group in our study. On the other hand, it would have been difficult to obtain red coloured edible oil which tastes similar to RPO. Since *β*-carotene supplementation with supplement pills in CF patients and *β*-carotene supplementation with RPO in non-CF subjects were shown to be successful, we forwent a control group. Instead, the recruited CF patients served as their own control using their serum concentrations before intervention. Second, we had a relatively high dropout rate. However, CF patients are always on risk to suffer acute pulmonary exacerbations which led to exclusion from the study since this would have influenced their antioxidant status. Further, CF patients are advised to take high amounts of fat-containing food. It might be possible that inclusion of RPO in the daily diet was not as easy as thought before. These reasons may have contributed to the dropout of finally five of 16 CF patients participating in the study. Because these potential problems were anticipated, our recruitment goal was more than 15 CF patients to end up with at least 10 patients, as aimed for based on our sample size calculation. Third, we did not see a substantial increase of *β*-carotene or retinol in the BMC of our subjects. One reason may be seen in the generally low tissue values of antioxidants in CF patients. Alternatively, the duration of *β*-carotene supplementation was not long enough to demonstrate a beneficial effect in BMC. We, therefore, recommend longer intervention periods in future studies evaluating RPO supplementation in CF patients.

## 4. Conclusion

Enrichment of daily food with 2 to 3 tablespoons of RPO over eight weeks was sufficient to elevate *β*-carotene, retinol, and *α*-carotene plasma concentrations in CF patients. Supplementation of daily food with RPO is easy to achieve and did not have any adverse effects. Our pilot study therefore suggests that low *β*-carotene levels in CF patients may be elevated efficiently by consumption of RPO. Further studies are needed to determine (i) if this approach can be used to correct deficient retinol levels in CF patients and (ii) if food oils enriched with other micronutrients in the daily diet may be an approach to supplement other antioxidants.

## Supplementary Material

Carotenoid composition of crude red palm oil (Choo YM et al., Journal of the American Oil Chemists' Society, 1996)

## Figures and Tables

**Table 1:** Characteristics of CF patients beginning the study (*n* = 16) and of the subjects who completed the study and were eligible for evaluation (*n* = 11).

Patient's characteristics	Subjects entering the study (*n* = 16), mean ± SD (range)	Subjects completing the study (*n* = 11), mean ± SD (range)
Age (years)	16.3 ± 5.6	15.8 ± 6.4
Gender, % male	50	54
F508del/F508del	10	7
F508del/other CF causing mutation	6	4
Pancreatic insufficiency	16	11
Weight (kg)	48.3 ± 12.5	45.7 ± 13.3
Height (cm)	160.7 ± 13.7	157.6 ± 15.6
BMI (kg/m^2^)	18.4 ± 2.0	18.0 ± 1.9
FEV1 (% pred.)	67.6 ± 21.9	69.7 ± 21.1
Creatinine (mg/dL)	06. ± 0.1	0.6 ± 0.1
AST (units/L)	21.1 ± 6.2	22.1 ± 6.3
ALT (units/L)	21.7 ± 6.8	21.4 ± 6.0
Albumin (g/L)	43.0 ± 4.0	43.8 ± 3.0
IgG (g/L)	13.5 ± 4.4	13.5 ± 4.9

**Table 2:** Concentrations of *β*-carotene, lycopene, *α*-tocopherol, and vitamin C in buccal mucosa cells of 11 CF patients. Results are given in pmol/*µ*g DNA [CFP: CF patient, <DL: below detection limit, n.d.: not detectable].

	*β*-Carotene [pmol/*µ*g DNA]		Lycopene [pmol/*µ*g DNA]		*α*-Tocopherol [pmol/*µ*g DNA]		Vitamin C [pmol/*µ*g DNA]	
	Before	After	Before	After	Before	After	Before	After
CFP 1	n.d.	0.1	<DL	0.2	22.6	8.8	7.8	12.0
CFP 2	0.1	0.1	n.d.	n.d.	26.9	19.6	20.9	2.8
CFP 3	n.d.	<DL	<DL	n.d.	25.3	36.0	n.d.	n.d.
CFP 4	n.d.	0.1	0.0	0.2	12.2	21.7	9.8	5.0
CFP 5	0.1	0.1	n.d.	n.d.	6.4	5.5	3.7	4.7
CFP 6	0.1	0.3	n.d.	0.1	19.0	11.5	8.7	4.8
CFP 7	0.0	0.1	0.3	0.1	31.4	47.5	8.3	18.0
CFP 8	0.1	n.d.	0.4	0.5	14.7	14.5	5.4	1.6
CFP 9	<DL	<DL	0.0	0.0	11.6	9.5	8.5	<DL
CFP 10	n.d.	0.2	n.d.	0.0	n.d.	13.1	n.d.	<DL
CFP 11	0.1	n.d.	0.1	n.d.	16.4	7.7	10.0	2.3

## Abbreviations

ALT: Alanine transaminase
AST: Aspartate transaminase
BMC: Buccal mucosa cells
CF: Cystic fibrosis
FEV1: Forced expiratory volume in 1 second
LPO: Lipid peroxidation
PI: Pancreatic insufficiency
RPO: Red palm oil.

## References

[1] Sies H., Stahl W. (1995). Vitamins E and C, *β*-carotene, and other carotenoids as antioxidants. *The American Journal of Clinical Nutrition*.

[2] Sokol R. J., Reardon M. C., Accurso F. J. (1989). Fat-soluble-vitamin status during the first year of life in infants with cystic fibrosis identified by screening of newborns. *The American Journal of Clinical Nutrition*.

[3] Range S. P., Dunster C., Knox A. J., Kelly F. J. (1999). Treatment of pulmonary exacerbations of cystic fibrosis leads to improved antioxidant status. *European Respiratory Journal*.

[4] McGrath L. T., Mallon P., Dowey L. (1999). Oxidative stress during acute respiratory exacerbations in cystic fibrosis. *Thorax*.

[5] Hartl D., Gaggar A., Bruscia E. (2012). Innate immunity in cystic fibrosis lung disease. *Journal of Cystic Fibrosis*.

[6] Mall M. A., Hartl D. (2014). CFTR: cystic fibrosis and beyond. *European Respiratory Journal*.

[7] Homnick D. N., Cox J. H., DeLoof M. J., Ringer T. V. (1993). Carotenoid levels in normal children and in children with cystic fibrosis. *Journal of Pediatrics*.

[8] Wood L. G., Fitzgerald D. A., Gibson P. G. (2002). Increased plasma fatty acid concentrations after respiratory exacerbations are associated with elevated oxidative stress in cystic fibrosis patients. *The American Journal of Clinical Nutrition*.

[9] Lancellotti L., D'Orazio C., Mastella G., Mazzi G., Lippi U. (1996). Deficiency of vitamins E and A in cystic fibrosis is independent of pancreatic function and current enzyme and vitamin supplementation. *European Journal of Pediatrics*.

[10] Borowitz D., Baker R. D., Stallings V. (2002). Consensus report on nutrition for pediatric patients with cystic fibrosis. *Journal of Pediatric Gastroenterology and Nutrition*.

[11] Sinaasappel M., Stern M., Littlewood J. (2002). Nutrition in patients with cystic fibrosis: a European Consensus. *Journal of Cystic Fibrosis*.

[12] Portal B. C., Richard M.-J., Faure H. S., Hadjian A. J., Favier A. E. (1995). Altered antioxidant status and increased lipid peroxidation in children with cystic fibrosis. *The American Journal of Clinical Nutrition*.

[13] Back E. I., Frindt C., Nohr D. (2004). Antioxidant deficiency in cystic fibrosis: when is the right time to take action?. *The American Journal of Clinical Nutrition*.

[14] Krinsky N. I. (1993). Actions of carotenoids in biological systems. *Annual Review of Nutrition*.

[15] Rust P., Eichler I., Renner S., Elmadfa I. (1998). Effects of long term oral beta-carotene supplementation on lipid peroxidation in patients with cystic fibrosis. *International Journal for Vitamin and Nutrition Research*.

[16] Renner S., Rath R., Rust P. (2001). Effects of *β*-carotene supplementation for six months on clinical and laboratory parameters in patients with cystic fibrosis. *Thorax*.

[17] Rust P., Eichler I., Renner S., Elmadfa I. (2000). Long-term oral *β*-carotene supplementation in patients with cystic fibrosis—effects on antioxidative status and pulmonary function. *Annals of Nutrition and Metabolism*.

[18] Oyewole O. E., Amosu A. M. (2010). Public health nutrition concerns on consumption of red palm-oil (RPO): the scientific facts from literature. *African Journal of Medicine and Medical Sciences*.

[19] Rice A. L., Burns J. B. (2010). Moving from efficacy to effectiveness: red palm oil's role in preventing vitamin A deficiency. *Journal of the American College of Nutrition*.

[20] Zeba A. N., Prével Y. M., Somé I. T., Delisle H. F. (2006). The positive impact of red palm oil in school meals on vitamin A status: study in Burkina Faso. *Nutrition Journal*.

[21] Lietz G., Henry C. J. K., Mulokozi G. (2001). Comparison of the effects of supplemental red palm oil and sunflower oil on maternal vitamin A status. *The American Journal of Clinical Nutrition*.

[22] Choo Y.-M., Yapa S.-C., Ooi C.-K., Ma A.-N., Goh S.-H., Ong A. S.-H. (1996). Recovered oil from palm-pressed fiber: a good source of natural carotenoids, vitamin E, and sterols. *Journal of the American Oil Chemists' Society*.

[23] Institute of Medicine (2001). *Dietary Reference Intakes for Vitamin A, Vitamin K, Arsenic, Boron, Chromium, Copper, Iodine, Manganese, Molybdenum, Nickel, Silicon, Vanadium, and Zinc*.

[24] Stahl W., Sundquist A. R., Hanusch M., Schwarz W., Sies H. (1993). Separation of *β*-carotene and lycopene geometrical isomers in biological samples. *Clinical Chemistry*.

[25] Polidori M. C., Stahl W., Eichler O., Niestroj I., Sies H. (2001). Profiles of antioxidants in human plasma. *Free Radical Biology & Medicine*.

[26] Urbain P., Raynor A., Bertz H., Lambert C., Biesalski H.-K. (2012). Role of antioxidants in buccal mucosa cells and plasma on the incidence and severity of oral mucositis after allogeneic haematopoietic cell transplantation. *Supportive Care in Cancer*.

[27] Natarajan N., Shambaugh G. E., Elseth K. M., Haines G. K., Radosevich J. A. (1994). Adaptation of the diphenylamine (DPA) assay to a 96-well plate tissue culture format and comparison with the MTT assay. *BioTechniques*.

[28] Winklhofer-Roob B. M., Puhl H., Khoschsorur G., Van't Hof M. A., Esterbauer H., Shmerling D. H. (1995). Enhanced resistance to oxidation of low density lipoproteins and decreased lipid peroxide formation during beta-carotene supplementation in cystic fibrosis. *Free Radical Biology and Medicine*.

[29] Lietz G., Mulokozi G., Henry J. C. K., Tomkins A. M. (2006). Xanthophyll and hydrocarbon carotenoid patterns differ in plasma and breast milk of women supplemented with red palm oil during pregnancy and lactation. *Journal of Nutrition*.

[30] Albanes D., Virtamo J., Taylor P. R. (1997). Effects of supplemental beta-carotene, cigarette smoking, and alcohol consumption on serum carotenoids in the Alpha-Tocopherol, Beta-Carotene Cancer Prevention study. *The American Journal of Clinical Nutrition*.

[31] Gaziano J. M., Johnson E. J., Russell R. M. (1995). Discrimination in absorption or transport of beta-carotene isomers after oral supplementation with either all-frans- or 9-cis-beta-carotene. *The American Journal of Clinical Nutrition*.

[32] Sommerburg O., Siems W., Kraemer K. (2013). *Carotenoids and Vitamin A in Translational Medicine*.

[33] Schupp C., Olano-Martin E., Gerth C., Morrissey B. M., Cross C. E., Werner J. S. (2004). Lutein, zeaxanthin, macular pigment, and visual function in adult cystic fibrosis patients. *The American Journal of Clinical Nutrition*.

[34] Sommerburg O., Siems W. G., Hurst J. S., Lewis J. W., Kliger D. S., van Kuijk F. J. G. M. (1999). Lutein and zeaxanthin are associated with photoreceptors in the human retina. *Current Eye Research*.

[35] Bone R. A., Landrum J. T., Friedes L. M. (1997). Distribution of lutein and zeaxanthin stereoisomers in the human retina. *Experimental Eye Research*.

