# Supplementary material for: Supplementation with Red Palm Oil Increases β-Carotene and Vitamin A Blood Levels in Patients with Cystic Fibrosis
Source: Mediators Inflamm. 2015 Jan 26;2015:817127. doi: 10.1155/2015/817127 (PMC4321850; doi:10.1155/2015/817127)
Supplement: Supplementary file 1 — Carotenoid composition of crude red palm oil (Choo YM et al., Journal of the American Oil Chemists' Society, 1996) [file 817127.f1.pdf]

Table E1:

Carotinoid composition of crude red palm oil (Choo YM et al. J Am Oil Chem Soc 1996).

**Carotenoid**	**Red Palm Oil [mg/kg]**
**phytoene**	1.3
**phytofluene**	0.1
**cis-β-carotene**	0.7
**β-carotene**	56.0
**α-carotene**	35.1
**cis-α-carotene**	2.5
**υ-carotene**	0.7
**ξ-carotene**	0.3
**δ-carotene**	0.8
**neurosporene**	0.3
**β-zeaxanthin**	0.7
**α-zeacarotene**	0.2
**lycopene**	1.3
